# Cryo-EM model validation recommendations based on outcomes of the 2019 EMDataResource challenge

**DOI:** 10.1038/s41592-020-01051-w

**Published:** 2021-02-04

**Authors:** Catherine L. Lawson, Andriy Kryshtafovych, Paul D. Adams, Pavel V. Afonine, Matthew L. Baker, Benjamin A. Barad, Paul Bond, Tom Burnley, Renzhi Cao, Jianlin Cheng, Grzegorz Chojnowski, Kevin Cowtan, Ken A. Dill, Frank DiMaio, Daniel P. Farrell, James S. Fraser, Mark A. Herzik, Soon Wen Hoh, Jie Hou, Li-Wei Hung, Maxim Igaev, Agnel P. Joseph, Daisuke Kihara, Dilip Kumar, Sumit Mittal, Bohdan Monastyrskyy, Mateusz Olek, Colin M. Palmer, Ardan Patwardhan, Alberto Perez, Jonas Pfab, Grigore D. Pintilie, Jane S. Richardson, Peter B. Rosenthal, Daipayan Sarkar, Luisa U. Schäfer, Michael F. Schmid, Gunnar F. Schröder, Mrinal Shekhar, Dong Si, Abishek Singharoy, Genki Terashi, Thomas C. Terwilliger, Andrea Vaiana, Liguo Wang, Zhe Wang, Stephanie A. Wankowicz, Christopher J. Williams, Martyn Winn, Tianqi Wu, Xiaodi Yu, Kaiming Zhang, Helen M. Berman, Wah Chiu

**Affiliations:** 1 Institute for Quantitative Biomedicine, Rutgers, The State University of New Jersey, Piscataway, NJ USA; 2 Genome Center, University of California, Davis, CA USA; 3 Molecular Biophysics and Integrated Bioimaging Division, Lawrence Berkeley National Laboratory, Berkeley, CA USA; 4 Department of Bioengineering, University of California Berkeley, Berkeley, CA USA; 5 Department of Biochemistry and Molecular Biology, The University of Texas Health Science Center at Houston, Houston, TX USA; 6 Department of Integrated Computational Structural Biology, The Scripps Research Institute, La Jolla, CA USA; 7 York Structural Biology Laboratory, Department of Chemistry, University of York, York, UK; 8 Scientific Computing Department, UKRI Science and Technology Facilities Council, Research Complex at Harwell, Didcot, UK; 9 Department of Computer Science, Pacific Lutheran University, Tacoma, WA USA; 10 Department of Electrical Engineering and Computer Science, University of Missouri, Columbia, MO USA; 11 European Molecular Biology Laboratory, c/o DESY, Hamburg, Germany; 12 Laufer Center, Stony Brook University, Stony Brook, NY USA; 13 Department of Biochemistry and Institute for Protein Design, University of Washington, Seattle, WA USA; 14 Department of Bioengineering and Therapeutic Sciences, University of California San Francisco, San Francisco, CA USA; 15 Department of Chemistry and Biochemistry, University of California, San Diego, La Jolla, CA USA; 16 Department of Computer Science, Saint Louis University, St. Louis, MO USA; 17 Los Alamos National Laboratory, Los Alamos, NM USA; 18 Theoretical and Computational Biophysics, Max Planck Institute for Biophysical Chemistry, Göttingen, Germany; 19 Department of Biological Sciences, Purdue University, West Lafayette, IN USA; 20 Department of Computer Science, Purdue University, West Lafayette, IN USA; 21 Verna and Marrs McLean Department of Biochemistry and Molecular Biology, Baylor College of Medicine, Houston, TX USA; 22 Biodesign Institute, Arizona State University, Tempe, AZ USA; 23 School of Advanced Sciences and Languages, VIT Bhopal University, Bhopal, India; 24 The European Bioinformatics Institute (EMBL-EBI), Wellcome Genome Campus, Hinxton, UK; 25 Department of Chemistry, University of Florida, Gainesville, FL USA; 26 Division of Computing & Software Systems, University of Washington, Bothell, WA USA; 27 Department of Bioengineering, Stanford University, Stanford, CA USA; 28 Department of Biochemistry, Duke University, Durham, NC USA; 29 Structural Biology of Cells and Viruses Laboratory, Francis Crick Institute, London, UK; 30 Institute of Biological Information Processing (IBI-7: Structural Biochemistry) and Jülich Centre for Structural Biology (JuStruct), Forschungszentrum Jülich, Jülich, Germany; 31 Division of CryoEM and Biomaging, SSRL, SLAC National Accelerator Laboratory, Stanford University, Menlo Park, CA USA; 32 Physics Department, Heinrich Heine University Düsseldorf, Düsseldorf, Germany; 33 Center for Development of Therapeutics, Broad Institute of MIT and Harvard, Cambridge, MA USA; 34 New Mexico Consortium, Los Alamos, NM USA; 35 Department of Biological Structure, University of Washington, Seattle, WA USA; 36 Biophysics Graduate Program, University of California, San Francisco, CA USA; 37 Department of Electrical Engineering and Computer Science, University of Missouri, Columbia, MO USA; 38 SMPS, Janssen Research and Development, Spring House, PA USA; 39 Department of Chemistry and Chemical Biology, Rutgers, The State University of New Jersey, Piscataway, NJ USA; 40 Department of Biological Sciences and Bridge Institute, University of Southern California, Los Angeles, CA USA

**Keywords:** Cryoelectron microscopy, Statistical methods, Protein databases, Proteins

## Abstract

This paper describes outcomes of the 2019 Cryo-EM Model Challenge. The goals were to (1) assess the quality of models that can be produced from cryogenic electron microscopy (cryo-EM) maps using current modeling software, (2) evaluate reproducibility of modeling results from different software developers and users and (3) compare performance of current metrics used for model evaluation, particularly Fit-to-Map metrics, with focus on near-atomic resolution. Our findings demonstrate the relatively high accuracy and reproducibility of cryo-EM models derived by 13 participating teams from four benchmark maps, including three forming a resolution series (1.8 to 3.1 Å). The results permit specific recommendations to be made about validating near-atomic cryo-EM structures both in the context of individual experiments and structure data archives such as the Protein Data Bank. We recommend the adoption of multiple scoring parameters to provide full and objective annotation and assessment of the model, reflective of the observed cryo-EM map density.

## Abstract (web summary)

A multi-laboratory study in the form of a community challenge assesses the quality of models that can be produced from cryo-EM maps using different software tools, the reproducibility of models generated by different users and the performance of metrics used for model validation.

## Main

Cryo-EM has emerged as a key method to visualize and model biologically important macromolecules and cellular machines. Researchers can now routinely achieve resolutions better than 4 Å, yielding new mechanistic insights into cellular processes and providing support for drug discovery^1^.

The recent explosion of cryo-EM structures raises important questions. What are the limits of interpretability given the quality of maps and resulting models? How can model accuracy and reliability be quantified under the simultaneous constraints of map density and chemical rules?

**Fig. 1:**
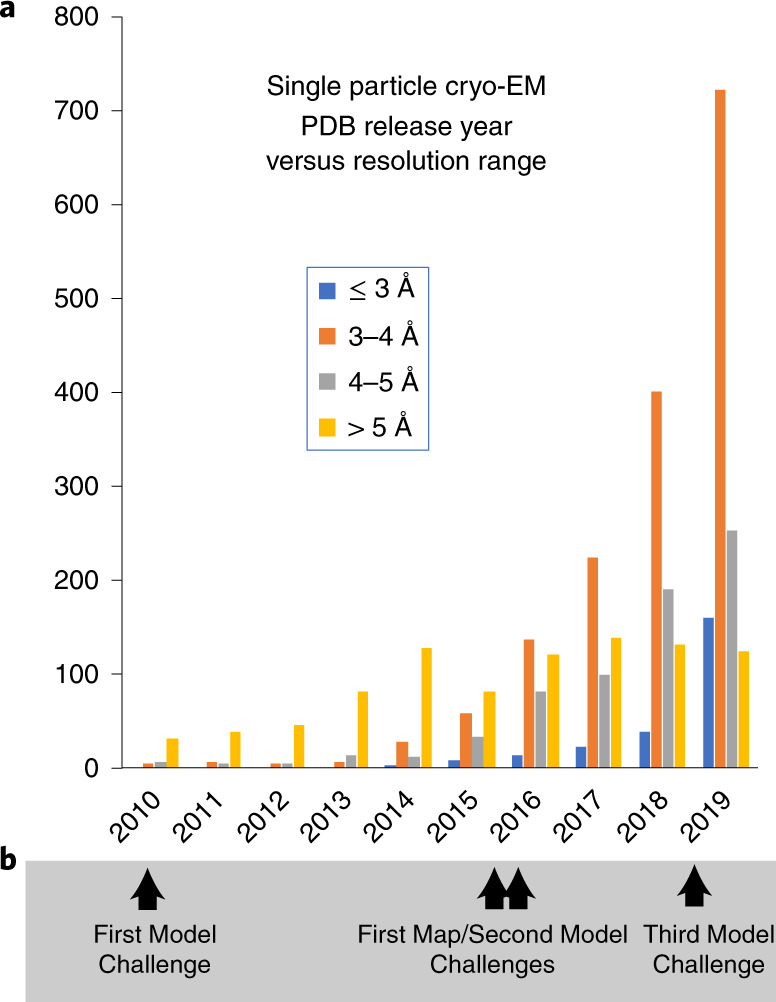
Single particle cryo-EM models in the Protein Data Bank. **a**, Plot of reported resolution versus PDB release year. Models derived from single particle cryo-EM maps have increased dramatically since the ‘resolution revolution’ circa 2014. Higher-resolution structures (blue bars) are also trending upward. **b**, EMDataResource challenge activities timeline. Source data

The EMDataResource Project (EMDR) (emdataresource.org) aims to derive validation methods and standards for cryo-EM structures through community consensus^2^. EMDR has convened an EM Validation Task Force^3^ analogous to those for X-ray crystallography^4^ and NMR^5^ and has sponsored challenges, workshops and conferences to engage cryo-EM experts, modelers and end-users^2,6^. During this period, cryo-EM has evolved rapidly (Fig. 1).

This paper describes outcomes of EMDR’s most recent challenge, the 2019 Model ‘Metrics’ Challenge. Map targets representing the state-of-the-art in cryo-EM single particle reconstruction were selected in the near-atomic resolution regime (1.8–3.1 Å) with a twist: three form a resolution series from the same specimen/imaging experiment. Careful evaluation of submitted models by participating teams leads us to several specific recommendations for validating near-atomic cryo-EM structures, directed toward both individual researchers and the Protein Data Bank (PDB) structure data archive^7^.

## Results

### Challenge design

**Fig. 2:**
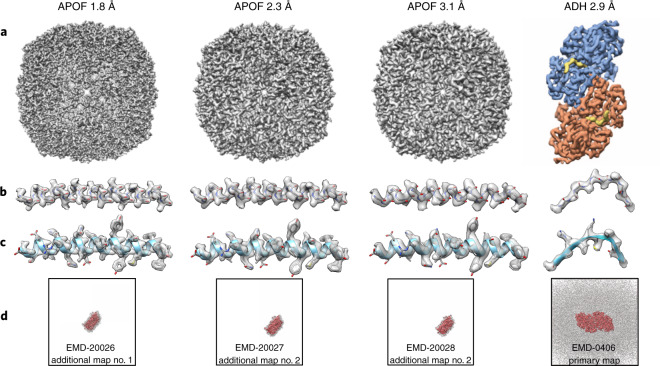
Challenge targets: cryo-EM maps at near-atomic resolution. Shown from left to right are ɑ-helix-rich APOF at 1.8, 2.3 and 3.1 Å (EMDB entries EMD-20026, EMD-20027 and EMD-20028) and ADH at 2.9 Å (EMDB entry EMD-0406). **a**, Full maps for each target. **b**,**c**, Representative secondary structural elements (APOF, residues 14–42; ADH, residues 34–45) with masked density for protein backbone atoms only (**b**), and for all protein atoms (**c**). Visible map features transition from near-atomic to secondary -structure dominated over the 1.8–3.1 Å resolution range. **d**, EMDB maps used in model Fit-to-Map analysis (APOF targets, masked single subunits; ADH, unmasked sharpened map). The molecular boundary is shown in red at the EMDB recommended contour level, background noise is represented in gray at one-third of the EMDB recommended contour level and the full map extent is indicated by the black outline.

Challenge targets (Fig. 2) consisted of a three-map human heavy-chain apoferritin (APOF) resolution series (a 500-kDa octahedral complex of 24 ɑ-helix-rich subunits), with maps differing only in the number of particles used in reconstruction^8^, plus a single map of horse liver alcohol dehydrogenase (ADH) (an 80-kDa ɑ/β homodimer with NAD and Zn ligands)^9^.

**Fig. 3:**
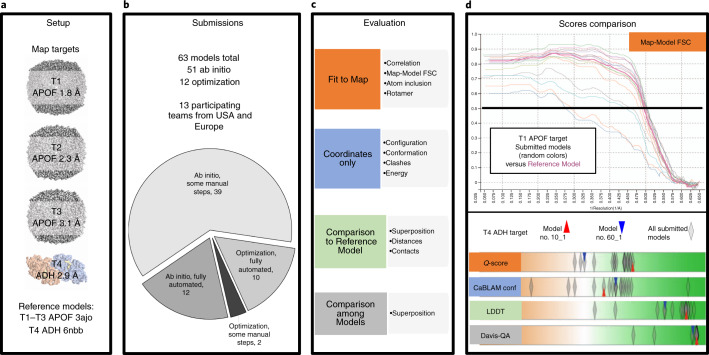
Challenge pipeline. **a**–**c**, Overview of the challenge setup (**a**), submissions (**b**) and evaluation (**c**) strategy. **d**, Scores comparison. Multiple interactive tabular and graphical displays enable comparative evaluations (model-compare.emdataresource.org). Top, Map-Model FSC curves, APOF 1.8 Å models (random light colors) versus reference model (bold cherry red). Map-Model FSC measures overall agreement of the experimental density map with a density map derived from the coordinate model (model map)^12^. Curves are calculated from Fourier coefficients of the two maps and plotted versus frequency (resolution^−1^). The resolution value corresponding to FSC = 0.5 (black horizontal line) is typically reported. Smaller values indicate better fit. Bottom, scores comparison tool, ADH models. Interactive score distribution sliders reveal at a glance how well submitted models performed relative to each other. Parallel lanes display score distributions for each evaluated metric in a manner conceptually similar to the graphical display for key metrics used in wwPDB validation reports^4,32^. Score distributions are shown for four representative metrics, one from each evaluation track. Model scores are plotted horizontally (semi-transparent diamonds) with color coding to indicate worse (left, orange) and better (right, green) values. Darker, opaque diamonds indicate multiple overlapping scores. Scores for two individual models are also highlighted: the interactive display enables individual models to be identified and compared (red and blue triangles).

A key criterion for target selection was availability of high-quality, experimentally determined model coordinates to serve as references (Fig. 3a). A 1.5 Å X-ray structure^10^ served as the APOF reference since no cryo-EM model was available. The X-ray model provides an excellent although not a fully optimized fit to each map, owing to method/sample differences. For ADH, the structure deposited by the original cryo-EM study authors served as the reference^9^.

Thirteen teams from the USA and Europe submitted 63 models in total, using whatever modeling software they preferred, yielding 15–17 submissions per target (Fig. 3b and Table 1). Most (51) were created ab initio, sometimes supported by additional manual steps, while others (12) were optimizations of publicly available models. The estimated human effort per model was 7 h on average, with a wide range (0–80 h).

**Table 1:** Participating modeling teams

Team ID^a^, name	Team members	No. of submitted models	Effort type(s)	Software
10 Yu	X. Yu	4	ab initio+manual	Phenix^21^, Buccaneer^37^, Chimera^38^, Coot^29^, Pymol
25 Cdmd	M. Igaev, A. Vaiana, H. Grubmüller	4	optimization automated	CDMD^39^
27 Kumar	D. Kumar	1	ab initio+manual	Phenix, Rosetta^40^, Buccaneer, ARP/wARP^41^, Coot
28 Ccpem	S. W. Hoh, K. Cowtan, A. P. Joseph, C. Palmer, M. Winn, T. Burnley, M. Olek, P. Bond, E. Dodson	4	ab initio+manual	CCPEM^42^, Refmac^13^, Buccaneer, Coot, TEMPy^16–18^
35 Phenix	P. Afonine, T. Terwilliger, L.-W. Hung	4	ab initio+manual	Phenix, Coot
38 Fzjuelich	G. Schroeder, L. Schaefer	3	optimization automated	Phenix, Chimera, DireX^43^, MDFF^44^, CNS, Gromacs
41 Arpwarp	G. Chojnowski	8	ab initio automated, ab initio+manual	Refmac, ARP/wARP, Coot
54 Kihara	D. Kihara, G. Terashi	8	ab initio+manual	Rosetta, Mainmast^45^, MDFF, Chimera
60 Deeptracer	L. Wang, D. Si, R. Cao, J. Cheng, S. A. Moritz, J. Pfab, T. Wu, J. Hou	10	ab initio automated, ab initio+manual	Cascaded-CNN^46^, Chimera
73 Singharoy	M. Shekhar, G. Terashi, S. Mittal, D. Sarkar, D. Kihara, K. Dill, A. Perez, A. Singharoy	5	ab initio+manual, optimization automated	reMDFF^47^, MELD^48^, VMD, Chimera, Mainmast
82 Rosetta	F. DiMaio, D. Farrell	8	ab initio automated, ab initio+manual	Rosetta, Chimera
90 Mbaker	M. Baker	2	ab initio+manual	Pathwalker^49^, Phenix, Chimera, Coot
91 Chiu	G. Pintilie, W. Chiu	2	optimization+manual	Phenix, Chimera, Coot

^a^Each team was assigned a random two-digit ID for blinded identification.

Submitted models were evaluated as in the previous challenge^11^ with multiple metrics in each of four tracks: Fit-to-Map, Coordinates-only, Comparison-to-Reference and Comparison-among-Models (Fig. 3c). The metrics include many in common use as well as several recently introduced.

Metrics to evaluate global Fit-to-Map included Map-Model Fourier shell correlation (FSC)^12^, FSC average^13^, Atom Inclusion^14^, EMRinger^15^, density-based correlation scores from TEMPy^16–18^, Phenix^19^ and the recently introduced *Q*-score to assess atom resolvability^8^.

Metrics to evaluate overall Coordinates-only quality included Clashscore, Rotamer outliers and Ramachandran outliers from MolProbity^20^, as well as standard geometry measures (for example, bond, chirality, planarity) from Phenix^21^. PDB currently uses all of these validation measures based on community recommendations^3–5^. New to this challenge round was CaBLAM, which evaluates protein backbone conformation using virtual dihedral angles^22^.

Metrics assessing similarity of model to reference included Global Distance Test^23^, Local Difference Distance Test^24^, CaRMSD^25^ and Contact Area Difference^26^. Davis-QA was used to measure similarity among submitted models^27^. These measures are widely used in critical assessment of protein structure prediction (CASP) competitions^27^.

Several metrics were also evaluated per residue. These were Fit-to-Map: EMRinger^15^, *Q*-score^8^, Atom Inclusion^14^, SMOC^18^ and CCbox^19^; and for Coordinates-only: Clashes, Ramachandran outliers^20^ and CaBLAM^22^.

Evaluated metrics are tabulated with brief definitions in Table 2 and extended descriptions are provided in Methods.

**Table 2:** Evaluated metrics

Metric class	Package metric definition
**Fit-to-Map**	
Correlation Coefficient, all voxels	Phenix **CCbox** full grid map versus model-map density correlation coefficient^19^ TEMPy **CCC** full grid map versus model-map density correlation coefficient^17^
Correlation Coefficient, selected voxels	Phenix **CCmask** map versus model-map density, only modeled regions^19^ Phenix **CCpeaks** map versus model-map density, only high-density map and model regions^19^ TEMPy **CCC_OV** map versus model-map density, overlapping map and model regions^18^ TEMPy **SMOC** Segment Manders’ Overlap, map versus model-map density, only modeled regions^18^
Correlation Coefficient, other density function	TEMPy **LAP** map versus model-map Laplacian filtered density (partial second derivative)^16^ TEMPy **Mutual Information** (MI) map versus model-map Mutual Information entropy-based function^16^ TEMPy **MI_OV** map versus model-map Mutual Information, only modeled regions^18^
Correlation Coefficient, atom positions	Chimera/MAPQ ***Q*-score** map density at each modeled atom versus reference Gaussian density function^8^
FSC	Phenix **FSC05** Resolution (distance) of Map-Model FSC curve read at point FSC = 0.5 (ref. ^19^) CCPEM/Refmac **FSCavg** FSC curve area integrated to map resolution limit^13,42^
Atom Inclusion	EMDB/VisualAnalysis **AI all** Atom Inclusion, percentage of atoms inside depositor-provided density threshold^14^ TEMPy **ENV** Atom Inclusion in envelope corresponding to sample molecular weight; penalizes unmodeled regions^16^
Sidechain Density	Phenix **EMRinger** evaluates backbone by sampling map density around Cɣ-atom ring paths for nonbranched residues^15^
**Coordinates-only**	
Configuration	Phenix **Bond** r.m.s.d. of bond lengths^21^ Phenix **Angle** r.m.s.d. of bond angles^21^ Phenix **Chiral** r.m.s.d. of chiral centers^21^ Phenix **Planar** r.m.s.d. of planar group planarity^21^ Phenix **Dihedral** r.m.s.d. of dihedral angles^21^
Clashes	MolProbity **Clashscore** Number of steric overlaps ≥0.4 Å per 1,000 atoms^20^
Conformation	MolProbity **Rotamer** sidechain conformation outliers^20^ MolProbity **Rama** Ramachandran *ɸ*,*ψ* main chain conformation outliers^20^ MolProbity **CaBLAM** outliers CO and Cɑ-based virtual dihedrals^22^ MolProbity **Calpha** outliers Cɑ-based virtual dihedrals and Cɑ virtual bond angle^22^
**Versus Reference Model**	
Atom Superposition	Local Global Alignment (LGA) **GDT-TS** Global Distance Test Total Score, average percentage of model Cɑ that superimpose with reference Cɑ, multiple distance cutoffs^23^ LGA **GDC** Global Distance Calculation, average percentage of all model atoms that superimpose with reference, multiple distance cutoffs^23^ LGA **GDC-SC** Global Distance Calculation for sidechain atoms only^23^ OpenStructure/QS **CaRMSD** r.m.s.d. of Cɑ atoms^25^
Interatomic Distances	LDDT **LDDT** Local Difference Distance Test, superposition-free comparison of all-atom distance maps between model and reference^24^
Contact Area	CAD **CAD** Contact Area Difference, superposition-free measure of differences in interatom contacts^26^ HBPLUS^50^ **HBPR** **>** **6**, hydrogen bond precision, nonlocal. fraction of correctly placed hydrogen bonds in residue pairs with >6 separation in sequence
**Comparison among models**	
Atom Superposition, Multiple	**DAVIS-QA** average of pairwise LGA GDT-TS scores among submitted models^27^

An evaluation system website with interactive tables, plots and tools (Fig. 3d) was established to organize and enable analysis of the challenge results and make the results accessible to all participants (model-compare.emdataresource.org).

### Overall and local quality of models

Most submitted models scored well, landing in ‘acceptable’ regions in each of the evaluation tracks, and in many cases performing better than the associated reference structure that served as a control (Supplementary Fig. 1). Teams that submitted ab initio models reported that additional manual adjustment was beneficial, particularly for the two lower resolution targets.

**Extended Data Fig. 1:**
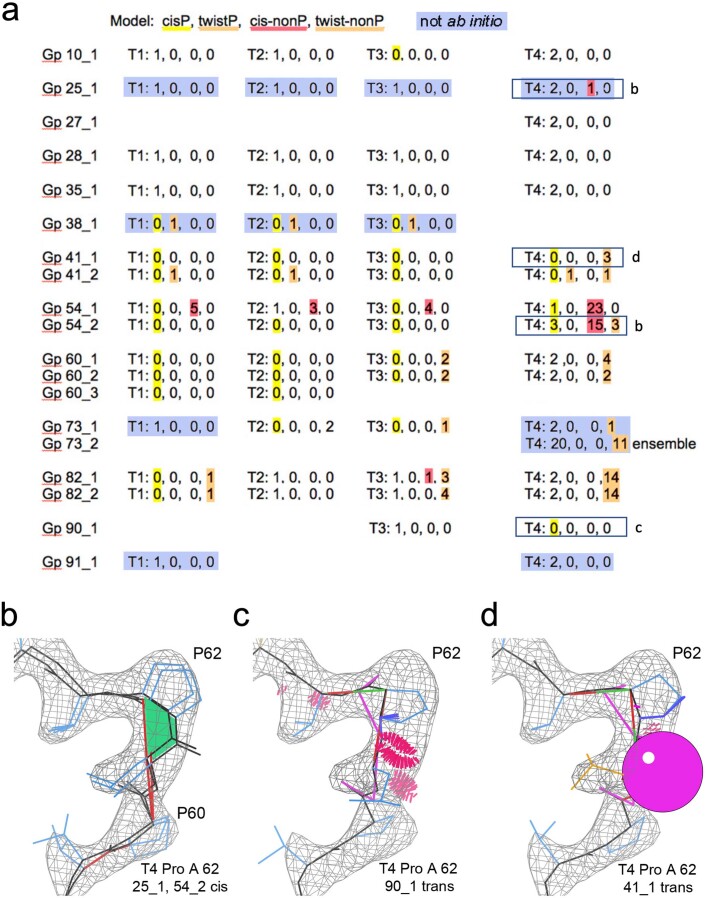
Evaluation of peptide bond geometry. All 63 Challenge models were evaluated using MolProbity. APOF and ADH each have one *cis* peptide bond per subunit before a proline residue. (**a**) Counts of peptide bonds with each of the following conformational properties: cisP: *cis* peptide before proline, twistP: non-planar peptide (>30°) before proline, cis-nonP: *cis* peptide before non-proline, twist-nonP: non-planar peptide bond before non-proline. Incorrect cis-nonP usually occurred where the model was misfit (see Extended Data Figs. 2 and 3), while incorrect *cis* or *trans* Pro usually produced poor geometry. Values inconsistent with reference models are highlighted. Statistically, 1 in 20 proline residues are genuinely *cis*; only 1 in 3000 non-proline residues are genuinely *cis*, and strongly non-planar peptide bonds (>30°) are almost never genuine^28^. Models are identified by the submitting group (Gp #, group id as defined in Table 1), model number (some groups submitted multiple models), and Target (T1-T3: APOF, T4: ADH). Optimized models are shaded blue. Only two groups (28, 31) had all peptides correct for all 4 targets. Models illustrated in panels **b**-**d** are indicated by labeled boxes. (**b**) Correct *cis* peptide geometry for Pro A62 in two ADH models. (**c**) Incorrect trans peptide geometry, with huge clashes up to 1.25 Å overlap (clusters of hot pink spikes), 2 CaBLAM outliers (magenta CO dihedral lines), and poor density fit. (**d**) Incorrect trans peptide geometry, with huge 1.9 Å C_β_ deviation at Leu 61 (magenta ball) because of incorrect hand of Cα, and 2 CaBLAM outliers. Molecular graphics were generated using KiNG.

Evaluation exposed four fairly frequent issues: mis-assignment of peptide-bond geometry, misorientation of peptides, local sequence misalignment and failure to model associated ligands. Two-thirds of submitted models had one or more peptide-bond geometry errors (Extended Data Fig. 1).

**Extended Data Fig. 2:**
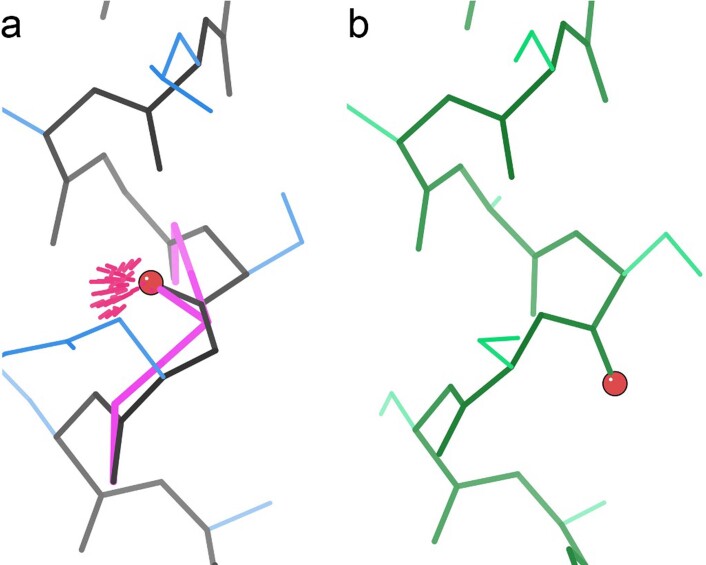
Classic CaBLAM outlier with no Ramachandran outlier. **a**, Mis-modeled peptide (identified by red ball at carbonyl oxgen position) is flagged by two successive CaBLAM outliers (magenta dihedrals), a bad clash (hot-pink spikes), and a bond-angle outlier (not shown), but no Ramachandran outlier. **b**, Correctly modeled peptide, involving a near-180° flip of the central peptide to achieve regular α-helical conformation. Ser 38 of T1/APOF model 60_1 is shown in (a); model 35_1 shown in (b). This example illustrates the most easily correctable situations: (1) for a CaBLAM outlier inside helix or β-sheet, regularize the secondary structure; (2) for two successive CaBLAM outliers, try flipping the central peptide. Molecular graphics were generated using KiNG. Note that sidechains are truncated by graphics clipping.

At resolutions near 3 Å or in weak local density, the carbonyl O protrusion disappears into the tube of backbone density (Fig. 2), and *trans* peptide bonds are more readily modeled in the wrong orientation. If peptide torsion *ϕ* (C,N,C_α_,C), *ψ* (N,C_α_,C,N) values are explicitly refined, adjacent sidechains can be pushed further in the wrong direction. Such cases are not flagged as Ramachandran outliers but they are recognized by CaBLAM^28^ (Extended Data Fig. 2).

**Extended Data Fig. 3:**
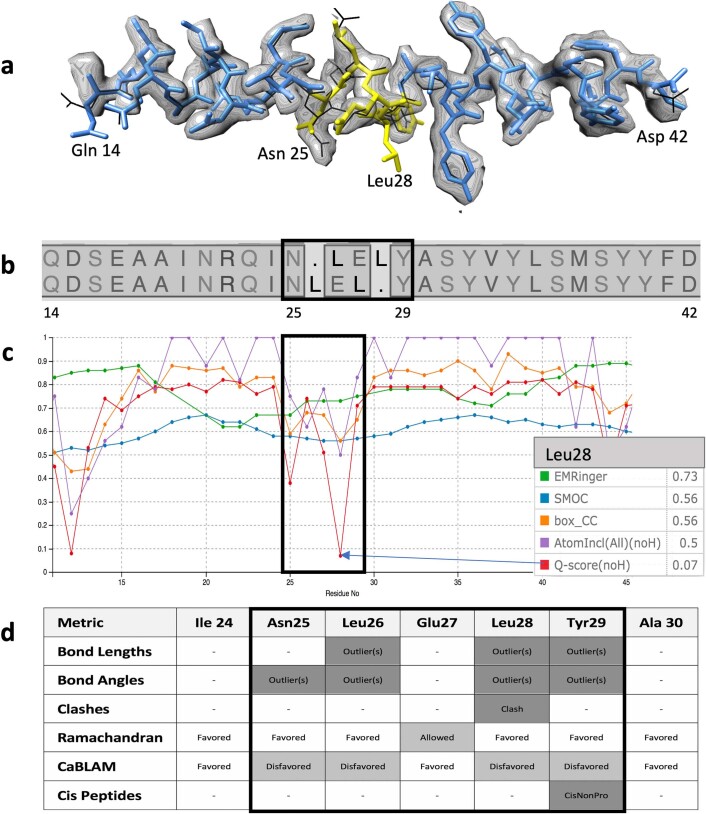
Evaluation of a short sequence misalignment within a helix. Local Fit-to-Map and Coordinates-only scores are compared for a 3-residue sequence misalignment inside an ɑ-helix in an *ab initio* model submitted to the Challenge (APOF 2.3 Å 54_1). **a**, Model residues 14–42 vs target map (blue: correctly placed residues, yellow: mis-threaded residues 25–29, black: APOF reference model, 3ajo). **b**, Structure-based sequence alignment of the *ab initio* model (top) vs. reference model (bottom). **c**, Local Fit-to-Map scores (screenshot from Challenge model evaluation website Fit-to-Map Local Accuracy tool). Curves are shown for Phenix Box_CC (orange), EMDB Atom Inclusion (purple), Q-score (red) EMRinger (green), and SMOC (blue). The score values for model residue Leu 28 are shown in the box at right. **d**, Residue scores were calculated using the Molprobity server. The mis-threaded region is boxed in (b-d). Panels (a) and (b) were generated using UCSF Chimera.

Sequence misthreadings misplace residues over very large distances. The misalignment can be recognized by local Fit-to-Map criteria, with ends flagged by CaBLAM, bad geometry, *cis*-nonPro peptides and clashes (Extended Data Fig. 3).

**Extended Data Fig. 4:**
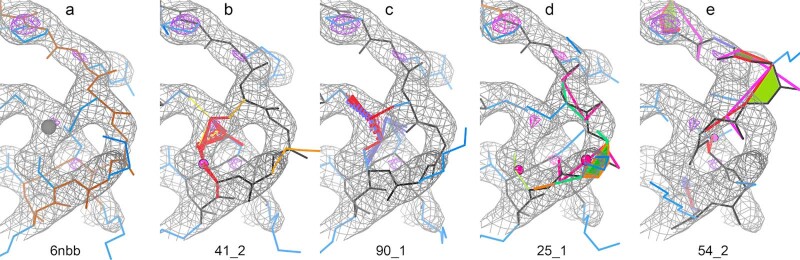
Modeling errors around omitted Zinc ligand in ADH. Target 4 (ADH) density map with examples of modeling errors caused by omission of Zinc ligand. **a**, Reference structure with Zinc metal ion (gray ball) coordinated by 4 Cysteine residues (blue sidechains). **b**-**e**, Submitted models missing Zinc (labels indicate the group_model ids). All have geometry and/or conformational violations as flagged by MolProbity CaBLAM (magenta pseudobonds), cis-nonPro (green parallelograms), Ramachandran (green pseudobonds), Cbeta (magenta spheres), and angle (blue and red fans). Model (**b**) has backbone conformation very close to correct, while (**b**) and (**c**) both have flags indicating bad geometry of incorrect disulfide bonds. Models (**c**) and (**d**) have backbone distortions, and (**e**) is mistraced through the Zn density. Molecular graphics were generated using KiNG.

ADH contains tightly bound ligands: an NADH cofactor as well as two zinc ions per subunit, with one zinc in the active site and the other in a spatially separate site coordinated by four cysteine residues^9^. Models lacking these ligands had considerable local modeling errors, sometimes even mistracing the backbone (Extended Data Fig. 4).

**Extended Data Fig. 5:**
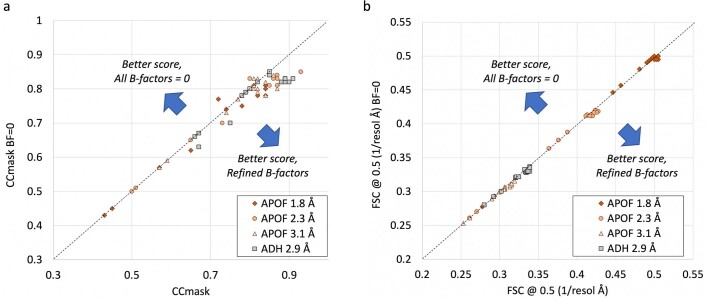
Fit-to-Map Scores with and without refined B-factors (ADP). Two representative metrics are shown: **a**, CCmask correlation, **b**, FSC05 resolution^−1^. Each plotted point indicates the calculated score for atom positions with B-factors included (horizontal axis) versus the calculated score for atom positions alone (vertical axis). Plot symbols identify map targets. Of 63 models total, 33 included refined B-factors. Differing scores +/- B-factors contribute off-diagonal points (black dotted lines are reference diagonals).

Although there was evidence for ordered water in higher-resolution APOF maps^8^, only two groups elected to model water. Submissions were also split roughly 50/50 for (1) inclusion of predicted H-atom positions and (2) refinement of isotropic B factors. Although near-atomic cryo-EM maps do not have a sufficient level of detail to directly identify H-atom positions, inclusion of predicted positions can still be useful for identifying steric properties such as H-bonds or clashes^20^. Where provided, refined B factors modestly improved Fit-to-Map scores (Extended Data Fig. 5).

### Evaluating metrics: Fit-to-Map

**Fig. 4:**
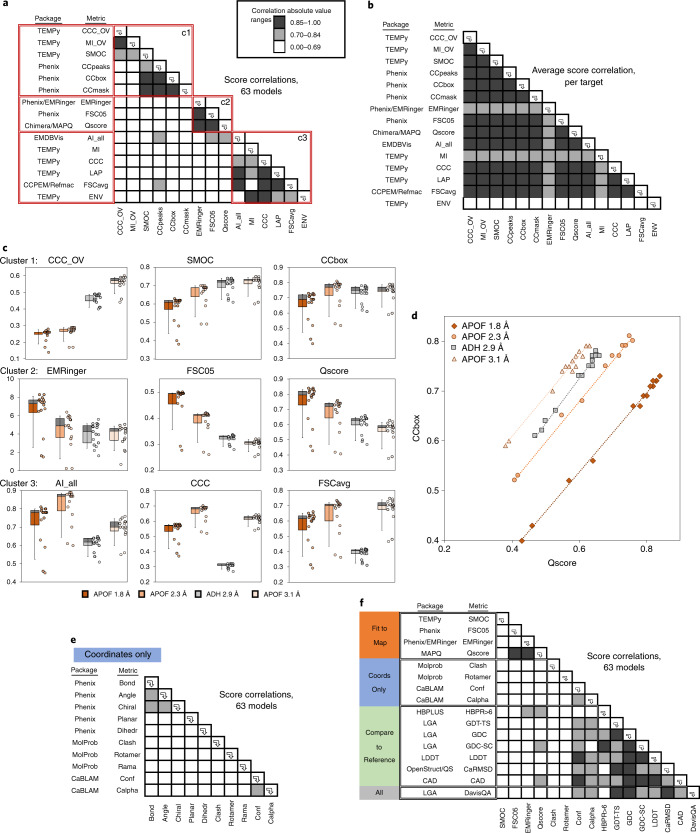
Evaluation of metrics. Model metrics (Table 2) were compared with each other to assess how similarly they performed in scoring the challenge models. **a**–**d**, Fit-to-Map metrics analyses. **a**, Pairwise correlations of scores for all models across all map targets (*n* = 63). **b**, Average correlation of scores per target (average over four correlation coefficients, one for each map target with T1, *n* = 16; T2, *n* = 15; T3, *n* = 15; T4, *n* = 17). Correlation-based metrics are identified by bold labels. In **a**, table order is based on a hierarchical cluster analysis (Methods). Three red-outlined boxes along the table diagonal correspond to identified clusters (no. c1–c3). For ease of comparison, order in **b** is identical to **a**. **c**, Representative score distributions are plotted by map target, ordered by map target resolution (see legend at bottom; T1, *n* = 16; T2, *n* = 15; T4, *n* = 17; T3, *n* = 15). Each row represents one of the three clusters defined in (a). Each score distribution is represented in box-and-whisker format (left) along with points for each individual score (right). Lower boxes represent Q1–Q2 (25th–50th percentile, in target color as shown in legend); upper boxes represent Q2–Q3 (25th–75th percentile, dark gray). Boxes do not appear when quartile limits are identical. Whiskers span 10th to 90th percentile. To improve visualization of closely clustered scores, individual scores (*y* values) are plotted against slightly dithered *x* values. **d**, Scores for one representative pair of metrics are plotted against each other (CCbox from cluster 1 and *Q*-score from Cluster 2). Diagonal lines represent linear fits by map target. **e**, Coordinates-only metrics comparison. **f**, Fit-to-Map, Coordinates-only and Comparison-to-Reference metrics comparison. Correlation levels in **a**,**b**,**e**,**f** are indicated by shading (see legend at top). See the Methods for additional details. Source data

Score distributions of Fit-to-Map metrics (Table 2) were systematically compared (Fig. 4a–d). For APOF, single subunits were evaluated against masked subunit maps, whereas for ADH, dimeric models were evaluated against the full sharpened cryo-EM map (Fig. 2d). To control for the varied impact of H-atom inclusion or isotropic *B*-factor refinement on different metrics, all evaluated scores were produced with H atoms removed and all B factors were set to zero.

Score distributions were first evaluated for all 63 models across all four challenge targets. A wide diversity in performance was observed, with poor correlations between most metrics (Fig. 4a). This means that a model that scored well relative to all 62 others using one metric may have a much poorer ranking using another. Hierarchical analysis identified three distinct clusters of similarly performing metrics (Fig. 4a, labels c1–c3).

The unexpected sparse correlations and clustering can be understood by considering per-target score distribution ranges, which differ substantially from each other. The three clusters identify sets of metrics that share similar trends (Fig. 4c).

Cluster 1 metrics (Fig. 4c, top row) share the trend of decreasing score values with increasing map resolution. The cluster consists of six real-space correlation measures, three from TEMPy^16–18^ and three from Phenix^19^. Each evaluates a model’s fit in a similar way: by correlating calculated model-map density with experimental map density. In most cases (five out of six), correlation is performed after model-based masking of the experimental map. This observed trend is contrary to the expectation that a Fit-to-Map score should increase as resolution improves. The trend arises at least in part because map resolution is an explicit input parameter for this class of metrics. For a fixed map/model pair, changing the input resolution value will change the score. As map resolution increases, the level of detail that a model-map must faithfully replicate to achieve a high correlation score must also increase.

Cluster 2 metrics (Fig. 4c, middle row) share the inverse trend: score values improve with increasing map target resolution. Cluster 2 metrics consist of Phenix Map-Model FSC = 0.5 (ref. ^19^), *Q*-score^8^ and EMRinger^15^. The observed trend is expected: by definition, each metric assesses a model’s fit to the experimental map in a manner that is intrinsically sensitive to map resolution. In contrast with cluster 1, cluster 2 metrics do not require map resolution to be supplied as an input parameter.

Cluster 3 metrics (Fig. 4c, bottom row) share a different overall trend: score values are substantially lower for ADH relative to APOF map targets. These measures include three unmasked correlation functions from TEMPy^16–18^, Refmac FSCavg^13^, Electron Microscopy Data Bank (EMDB) Atom Inclusion^14^ and TEMPy ENV^16^. All of these measures consider the full experimental map without masking, so can be sensitive to background noise, which is substantial in the unmasked ADH map and minimal in the masked APOF maps (Fig. 2d).

Score distributions were also evaluated for how similarly they performed per target, and in this case most metrics were strongly correlated with each other (Fig. 4b). This means that for any single target, a model that scored well relative to all others using one metric also fared well using nearly every other metric. This situation is illustrated by comparing scores for two different metrics, CCbox from cluster 1 and *Q*-score from cluster 2 (Fig. 4d). The plot’s four diagonal lines demonstrate that the scores are tightly correlated with each other within each map target. But, as described above, the two metrics have different sensitivities to map-specific factors. It is these different sensitivities that give rise to the separated, parallel spacings of the four diagonal lines, indicating score ranges on different relative scales.

One Fit-to-Map metric showed poor per-target correlation with all others: TEMPy ENV (Fig. 4b). ENV evaluates atom positions relative to a density threshold that is based on sample molecular weight. At near-atomic resolution this threshold is overly generous. TEMPy Mutual Information and EMRinger also diverged from others (Fig. 4b). Mutual information scores reflected strong influence of ADH background noise. In contrast, masked MI_OV correlated well with other measures. EMRinger yielded distinct distributions owing to its focus on backbone placement^15^.

Collectively these results reveal that multiple factors such as using experimental map resolution as an input parameter, presence of background noise and density threshold selection can strongly affect Fit-to-Map score values, depending on the chosen metric. These are not desirable features for archive-wide validation of deposited cryo-EM structures.

### Evaluating metrics: Coordinates-only and versus Reference

Metrics to assess model quality based on Coordinates-only (Table 2), as well as Comparison-to-Reference and Comparison-among-Models (Table 2) were also evaluated and compared (Fig. 4e,f).

Most Coordinates-only metrics were poorly correlated with each other (Fig. 4e), with the exception of bond, bond angle and chirality root mean squared deviation (r.m.s.d.), which form a small cluster. Ramachandran outliers, widely used to validate protein backbone conformation, were poorly correlated with all other Coordinates-only measures. More than half (33) of submitted models had zero Ramachandran outliers, while only four had zero CaBLAM conformation outliers. Ramachandran statistics are increasingly used as restraints^29,30^, which reduces their use as a validation metric. These results support the concept of CaBLAM as an informative score for validating backbone conformation^22^.

**Extended Data Fig. 6:**
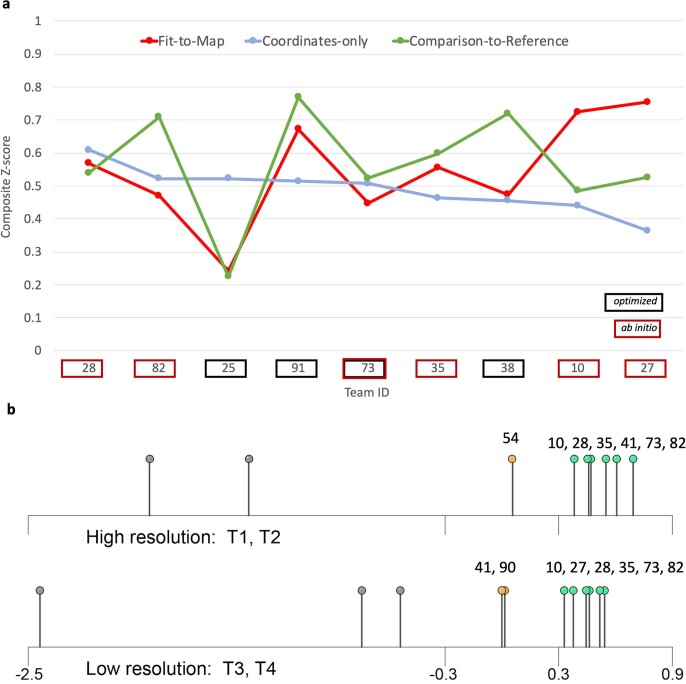
Group performance evaluations. **a**, Group composite Z-scores plotted by metric category. The nine teams with highest Coordinate-only composite Z-score rankings are shown, sorted left to right. The plot illustrates that by group/method, Coordinate-only scores are poorly corelated with Fit-to-Map and Comparison-to-Reference scores. In contrast, a modest correlation is observed between Fit-to-Map and Comparison-to-Reference scores. **b**, Averaged model composite Z-scores plotted for ab initio modeling groups at higher resolution (T1 at 1.8 Å, T2 at 2.3 Å) and lower resolution (T3 at 3.1 Å, T4 at 2.9 Å). In each case 6 groups produced very good models (Z ≥ 0.3; green pins), though not the same set. Runner-up clusters (−0.3 ≤ Z < 0.3) are shown with gold pins. Individual scores and order shift with alternate choices of evaluation metrics and weights, but the clusters at each resolution level are stable. Composite Z-scores were calculated as described in Methods.

CaBLAM metrics, while orthogonal to other Coordinates-only measures, were unexpectedly found to perform very similarly to Comparison-to-Reference metrics. The similarity likely arises because the worst modeling errors in this challenge were sequence and backbone conformation mis-assignments. These errors were equally flagged by CaBLAM, which compares models against statistics from high-quality PDB structures, and the Comparison-to-Reference metrics, which compare models against a high-quality reference. To a lesser extent, modeling errors were also flagged by Fit-to-Map metrics (Fig. 4f). Overall, Coordinates-only metrics were poorly correlated with Fit-to-Map metrics (Fig. 4f and Extended Data Fig. 6a).

Protein sidechain accuracy is specifically assessed by Rotamer and GDC-SC, while EMRinger, *Q*-score, CAD, hydrogen bonds in residue pairs (HBPR > 6), GDC and LDDT metrics include sidechain atoms. For these eight measures, Rotamer was completely orthogonal, *Q*-score was modestly correlated with the Comparison-to-Reference metrics, and EMRinger, which measures sidechain fit as a function of main chain conformation, was largely independent (Fig. 4f). These results suggest a need for multiple metrics (for example, *Q*-score, EMRinger, Rotamer) to assess different aspects of sidechain quality.

### Evaluating metrics: local scoring

Several residue-level scores were calculated in addition to overall scores. Five Fit-to-Map metrics considered masked density for both map and model around the evaluated residue (CCbox^19^, SMOC^18^), density profiles at nonhydrogen atom positions (*Q*-score^8^), density profiles of nonbranched residue Cɣ-atom ring paths (EMRinger^15^) or density values at non-H-atom positions relative to a chosen threshold (Atom Inclusion^14^). In two of these five, residue-level scores were obtained as sliding-window averages over multiple contiguous residues (SMOC, nine residues; EMRinger, 21 residues).

Residue-level correlation analyses similar to those described above (not shown) indicate that local Fit-to-Map scores diverged more than their corresponding global scores. Residue-level scoring was most similar across evaluated metrics for high resolution maps. This observation suggests that the choice of method for scoring residue-level fit becomes less critical at higher resolution, where maps tend to have stronger density/contrast around atom positions.

A case study of a local modeling error (Extended Data Fig. 3) showed that Atom Inclusion^14^, CCbox^19^ and *Q*-score^8^ produced substantially worse scores within a four-residue ɑ-helical misthread relative to correctly assigned flanking residues. In contrast, the sliding-window-based metrics were largely insensitive (a new TEMPy version offers single residue (SMOCd) and adjustable window analysis (SMOCf)^31^). At near-atomic resolution, single residue Fit-to-Map evaluation methods are likely to be more useful.

Residue-level Coordinates-only, Comparison-to-Reference and Comparison-among-Models metrics (not shown) were also evaluated for the same modeling error. The MolProbity server^20,22^ flagged the problematic four-residue misthread via CaBLAM, *cis*-Peptide, Clashscore, bond and angle scores, but all Ramachandran scores were either favored or allowed. The Comparison-to-Reference LDDT and LGA local scores and the Davis-QA model consensus score also strongly flagged this error. The example demonstrates the value of combining multiple orthogonal measures to identify geometry issues, and further highlights the value of CaBLAM as an orthogonal measure for backbone conformation.

### Group performance

Group performance was examined by modeling category and target by combining *Z*-scores from metrics determined to be meaningful in the analyses described above (Methods and Extended Data Fig. 6). A wide variety of map density features and algorithms were used to produce a model, and most were successful yet allowing a few mistakes, often in different places (Extended Data Figs. 1–4). For practitioners, it might be beneficial to combine models from several ab initio methods for subsequent refinement.

## Discussion

This third EMDR Model Challenge has demonstrated that cryo-EM maps with a resolution ≤3 Å and from samples with limited conformational flexibility have excellent information content, and automated methods are able to generate fairly complete models from such maps, needing only small amounts of manual intervention.

Inclusion of maps in a resolution series enabled controlled evaluation of metrics by resolution, with a completely different map providing a useful additional control. These target selections enabled observation of important trends that otherwise could have been missed. In a recent evaluation of predicted models in the CASP13 competition against several roughly 3 Å cryo-EM maps, TEMPy and Phenix Fit-to-Map correlation measures performed very similarly^31^. In this challenge, because the chosen targets covered a wider resolution range and had more variability in background noise, the same measures were found to have distinctive, map feature-sensitive performance profiles.

Most submitted models were overall either equivalent to or better than their reference model. This achievement reflects significant advances in the development of modeling tools relative to the state presented a decade ago in our first model challenge^2^. However, several factors beyond atom positions that become important for accurate modeling at near-atomic resolution were not uniformly addressed; only half included refinement of atomic displacement factors (B factors) and a minority attempted to fit water or bound ligands.

Fit-to-Map measures were found to be sensitive to different physical properties of the map, including experimental map resolution and background noise level, as well as input parameters such as density threshold. Coordinates-only measures were found to be largely orthogonal to each other and also largely orthogonal to Fit-to-Map measures, while Comparison-to-Reference measures were generally well correlated with each other.

The cryo-EM modeling community as represented by the challenge participants have introduced a number of metrics to evaluate models with sound biophysical basis. Based on our careful analyses of these metrics and their relationships, we make four recommendations regarding validation practices for cryo-EM models of proteins determined at near-atomic resolution as studied here between 3.1 and 1.8 Å, a rising trend for cryo-EM (Fig. 1a).

Recommendation 1. For researchers optimizing a model against a single map, nearly any of the evaluated global Fit-to-Map metrics (Table 2) can be used to evaluate progress because they are all largely equivalent in performance. The exception is TEMPy, ENV is more appropriate at lower resolutions (>4 Å).

Recommendation 2. To flag issues with local (per residue) Fit-to-Map, metrics that evaluate single residues are more suitable than those using sliding-window averages over multiple residues (Evaluating metrics: local scoring).

Recommendation 3. The ideal Fit-to-Map metric for archive-wide ranking will be insensitive to map background noise (appropriate masking or alternative data processing can help), will not require input of estimated parameters that affect score value (for example, resolution limit, threshold) and will yield overall better scores for maps with trustworthy higher-resolution features. The three cluster 2 metrics identified in this challenge (Fig. 4a ‘c2’ and Fig. 4c center row) meet these criteria.

- Map-Model FSC^12,19^ is already in common use, and can be compared with the experimental map’s independent half-map FSC curve.
- Global EMRinger score^15^ can assess nonbranched protein sidechains.
- *Q*-score can be used both globally and locally for validating nonhydrogen atom *x*,*y*,*z* positions^8^.

Other Fit-to-Map metrics may be rendered suitable for archive-wide comparisons through conversion of raw scores to *Z*-scores over narrow resolution bins, as is currently done by the PDB for some X-ray-based metrics^4,32^.

Recommendation 4. CaBLAM and MolProbity *cis*-peptide detection^22^ are useful to detect protein backbone conformation issues. These are particularly valuable tools for cryo-EM, since maps at typical resolutions (2.5–4.0 Å, Fig. 1a) may not resolve backbone carbonyl oxygens (Fig. 2).

In this challenge, more time could be devoted to analysis when compared with previous rounds because infrastructure for model collection, processing and assessment is now established. However, several important issues could not be addressed, including evaluation of overfitting using half-map based methods^13,33–35^, effect of map sharpening on Fit-to-Map scores^8,36^, validation of ligand fit and metal ion/water identification and validation at atomic resolution including H atoms. EMDR plans to sponsor additional model challenges to continue promoting development and testing of cryo-EM modeling and validation methods.

## Methods

### Challenge process and organization

Informed by previous challenges^2,6,11^, the 2019 Model Challenge process was substantially streamlined in this round. In March, a panel of advisors with expertise in cryo-EM methods, modeling and/or model assessment was recruited. The panel worked with EMDR team members to develop the challenge guidelines, identify suitable map targets from EMDB and reference models from the PDB and recommend the metrics to be calculated for each submitted model.

The challenge rules and guidance were as follows: (1) ab initio modeling is encouraged but not required. For optimization studies, any publicly available coordinate set can be used as the starting model. (2) Regardless of the modeling method used, submitted models should be as complete and as accurate as possible (that is, equivalent to publication-ready). (3) For each target, a separate modeling process should be used. (4) Fitting to either the unsharpened/unmasked map or one of the half-maps is strongly encouraged. (5) Submission in mmCIF format is strongly encouraged.

Members of cryo-EM and modeling communities were invited to participate in mid-April 2019 and details were posted on the challenges website (challenges.emdataresource.org). Models were submitted by participant teams between 1 and 28 May 2019. For APOF targets, coordinate models were submitted as single subunits at the position of a provided segmented density consisting of a single subunit. ADH models were submitted as dimers. For each submitted model, metadata describing the full modeling workflow were collected via a Drupal webform, and coordinates were uploaded and converted to PDBx/mmCIF format using PDBextract^51^. Model coordinates were then processed for atom/residue ordering and nomenclature consistency using PDB annotation software (Feng Z., https://sw-tools.rcsb.org/apps/MAXIT) and additionally checked for sequence consistency and correct position relative to the designated target map. Models were then evaluated as described below (Model evaluation system).

In early June, models, workflows and initial calculated scores were made available to all participants for evaluation, blinded to modeler team identity and software used. A 2.5-day workshop was held in mid-June at Stanford/SLAC to review the results, with panel members attending in person. All modeling participants were invited to attend remotely and present overviews of their modeling processes and/or assessment strategies. Recommendations were made for additional evaluations of the submitted models as well as for future challenges. Modeler teams and software were unblinded at the end of the workshop. In September, a virtual follow-up meeting with all participants provided an overview of the final evaluation system after implementation of recommended updates.

### Coordinate sources and modeling software

Modeling teams created ab initio models or optimized previously known models available from the PDB. Models optimized against APOF maps used PDB entries 2fha, 5n26 or 3ajo as starting models. Models optimized against ADH used PDB entries 1axe, 2jhf or 6nbb. Ab initio software included ARP/wARP^41^, Buccaneer^37^, Cascaded-CNN^46^, Mainmast^45^, Pathwalker^49^ and Rosetta^40^. Optimization software included CDMD^39^, CNS^52^, DireX^43^, Phenix^21^, REFMAC^13^, MELD^48^, MDFF^44^ and reMDFF^47^. Participants made use of VMD^53^, Chimera^38^, COOT^29^ and PyMol for visual evaluation and/or manual model improvement of map-model fit. See Table 1 for software used by each modeling team. Modeling software versions/websites are listed in the Nature Research Reporting Summary.

### Model evaluation system

The evaluation system for 2019 challenge (model-compare.emdataresource.org) was built on the basis of the 2016/2017 Model Challenge system^11^, updated with several additional evaluation measures and analysis tools. Submitted models were evaluated for >70 individual metrics in four tracks: Fit-to-Map, Coordinates-only, Comparison-to-Reference and Comparison-among-Models. A detailed description of the updated infrastructure and each calculated metric is provided as a help document on the model evaluation system website. Result data are archived at Zenodo^54^. Analysis software versions/websites are listed in the Nature Research Reporting Summary.

For brevity, a representative subset of metrics from the evaluation website are discussed in this paper. The selected metrics are listed in Table 2 and are further described below. All scores were calculated according to package instructions using default parameters.

#### Fit-to-Map

The evaluated metrics included several ways to measure the correlation between map and model density as implemented in TEMPy^16–18^ v.1.1 (CCC, CCC_OV, SMOC, LAP, MI, MI_OV) and the Phenix^21^ v.1.15.2 map_model_cc module^19^ (CCbox, CCpeaks, CCmask). These methods compare the experimental map with a model map produced on the same voxel grid, integrated either over the full map or over selected masked regions. The model-derived map is generated to a specified resolution limit by inverting Fourier terms calculated from coordinates, B factors and atomic scattering factors. Some measures compare density-derived functions instead of density (MI, LAP^16^).

The *Q*-score (MAPQ v.1.2 (ref. ^8^) plugin for UCSF Chimera^38^ v.1.11) uses a real-space correlation approach to assess the resolvability of each model atom in the map. Experimental map density is compared to a Gaussian placed at each atom position, omitting regions that overlap with other atoms. The score is calibrated by the reference Gaussian, which is formulated so that a highest score of 1 would be given to a well-resolved atom in a map at an approximately 1.5 Å resolution. Lower scores (down to −1) are given to atoms as their resolvability and the resolution of the map decreases. The overall *Q*-score is the average value for all model atoms.

Measures based on Map-Model FSC curve, Atom Inclusion and protein sidechain rotamers were also compared. Phenix Map-Model FSC is calculated using a soft mask and is evaluated at FSC = 0.5 (ref. ^19^). REFMAC FSCavg^13^ (module of CCPEM^42^) integrates the area under the Map-Model FSC curve to a specified resolution limit^13^. EMDB Atom Inclusion determines the percentage of atoms inside the map at a specified density threshold^14^. TEMPy ENV is also threshold-based and penalizes unmodeled regions^16^. EMRinger (module of Phenix) evaluates backbone positioning by measuring the peak positions of unbranched protein C_γ_ atom positions versus map density in ring paths around C_ɑ_–C_β_ bonds^15^.

#### Coordinates-only

Standard measures assessed local configuration (bonds, bond angles, chirality, planarity, dihedral angles; Phenix model statistics module), protein backbone (MolProbity Ramachandran outliers^20^; Phenix molprobity module) and sidechain conformations, and clashes (MolProbity rotamers outliers and Clashscore^20^; Phenix molprobity module).

New in this challenge round is CaBLAM^22^ (part of MolProbity and as Phenix cablam module), which uses two procedures to evaluate protein backbone conformation. In both cases, virtual dihedral pairs are evaluated for each protein residue *i* using C_ɑ_ positions *i* − 2 to *i* + 2. To define CaBLAM outliers, the third virtual dihedral is between the CO groups flanking residue *i*. To define Calpha-geometry outliers, the third parameter is the C_ɑ_ virtual angle at *i*. The residue is then scored according to virtual triplet frequency in a large set of high-quality models from PDB^22^.

#### Comparison-to-Reference and Comparison-among-Models

Assessing the similarity of the model to a reference structure and similarity among submitted models, we used metrics based on atom superposition (LGA GDT-TS, GDC and GDC-SC scores^23^ v.04.2019), interatomic distances (LDDT score^24^ v.1.2), and contact area differences (CAD^26^ v.1646). HBPLUS^50^ was used to calculate nonlocal hydrogen bond precision, defined as the fraction of correctly placed hydrogen bonds with more than six separations in sequence (HBPR > 6). DAVIS-QA determines for each model the average of pairwise GDT-TS scores among all other models^27^.

#### Local (per residue) scores

Residue-level visualization tools for comparing the submitted models were also provided for the following metrics: Fit-to-Map, Phenix CCbox, TEMPy SMOC, *Q*-score, EMRinger and EMDB Atom Inclusion; Comparison-to-Reference, LGA and LDDT; and Comparison-among-Models, DAVIS-QA.

### Metric score pairwise correlations and distributions

For pairwise comparisons of metrics, Pearson correlation coefficients (*P*) were calculated for all model scores and targets (*n* = 63). For average per-target pairwise comparisons of metrics, *P* values were determined for each target and then averaged. Metrics were clustered according to the similarity score (1 − |*P*|) using a hierarchical algorithm with complete linkage. At the beginning, each metric was placed into a cluster of its own. Clusters were then sequentially combined into larger clusters, with the optimal number of clusters determined by manual inspection. In the Fit-to-Map evaluation track, the procedure was stopped after three divergent score clusters were formed for the all-model correlation data (Fig. 4a), and after two divergent clusters were formed for the average per-target clustering (Fig. 4b).

### Controlling for model systematic differences

As initially calculated, some Fit-to-Map scores had unexpected distributions, owing to differences in modeling practices among participating teams. For models submitted with all atom occupancies set to zero, occupancies were reset to one and rescored. In addition, model submissions were split approximately 50/50 for each of the following practices: (1) inclusion of hydrogen atom positions and (2) inclusion of refined B factors. For affected fit-to-map metrics, modified scores were produced excluding hydrogen atoms and/or setting B factors to zero. Both original and modified scores are provided at the web interface. Only modified scores were used in the comparisons described here.

### Evaluation of group performance

Rating of group performance was done using the group ranks and model ranks (per target) tools on the challenge evaluation website. These tools permit users, either by group or for a specified target and for all or a subcategory of models (for example, ab initio), to calculate composite *Z*-scores using any combination of evaluated metrics with any desired relative weightings. The *Z*-scores for each metric are calculated from all submitted models for that target (*n* = 63). The metrics (weights) used to generate composite *Z*-scores were as follows.

#### Coordinates-only

CaBLAM outliers (0.5), Calpha-geometry outliers (0.3) and Clashscore (0.2). CaBLAM outliers and Calpha-geometry outliers had the best correlation with Comparison-to-Reference parameters (Fig. 4f), and Clashscore is an orthogonal measure. Ramachandran and rotamer criteria were excluded since they are often restrained in refinement and are zero for many models.

#### Fit-to-Map

EMRinger (0.3), *Q*-score (0.3), Atom Inclusion (0.2) and SMOC (0.2). EMRinger and *Q*-score were among the most promising model-to-map metrics, and the other two provide distinct measures.

#### Comparison-to-Reference

LDDT (0.9), GDC_all (0.9) and HBPR >6 (0.2). LDDT is superposition-independent and local, while GDC_all requires superposition; H-bonding is distinct. Metrics in this category are weighted higher, because although the reference models are not perfect, they are a reasonable estimate of the right answer.

Composite *Z*-scores by metric category (Extended Data Fig. 6a) used the Group Ranks tool. For ab initio rankings (Extended Data Fig. 6b), *Z*-scores were averaged across each participant group on a given target, and further averaged across T1 + T2 and across T3 + T4 to yield overall *Z*-scores for high and low resolutions group 54 models were rated separately because they used different methods. Group 73’s second model on target T4 was not rated because the metrics are not set up to meaningfully evaluate an ensemble. Other choices of metric weighting schemes were tried, with very little effect on clustering.

### Molecular graphics

Molecular graphics images were generated using UCSF Chimera^38^ (Fig. 2 and Extended Data Fig. 3) and KiNG^55^ (Extended Data Figs. 1, 2 and 4).

### Reporting Summary

Further information on research design is available in the Nature Research Reporting Summary linked to this article.

## Online content

Any methods, additional references, Nature Research reporting summaries, source data, extended data, supplementary information, acknowledgements, peer review information; details of author contributions and competing interests; and statements of data and code availability are available at 10.1038/s41592-020-01051-w.

## Supplementary information

Supplementary Information Supplementary Fig. 1.

Reporting Summary

## Data Availability

The map targets used in the challenge were downloaded from the EMDB, entries EMD-20026 (file emd_20026_additional_1.map.gz), EMD-20027 (file emd_20027_additional_2.map.gz), EMD-20028 (file emd_20028_additional_2.map.gz) and EMD-0406 (file emd_0406.map.gz). Reference models were downloaded from the PDB, entries 3ajo and 6nbb. Submitted models, model metadata, result logs and compiled data are archived at Zenodo at 10.5281/zenodo.4148789, and at https://model-compare.emdataresource.org/data/2019/. Interactive summary tables, graphical views and .csv downloads of compiled results are available at https://model-compare.emdataresource.org/2019/cgi-bin/index.cgi. Source data are provided with this paper.

## Source data

Source Data Fig. 1 Spreadsheet and graph, EM entries in PDB. Source Data Fig. 4 Spreadsheets and graphs, 2019 Model Metric Challenge results.
