# Supplementary material for: Cryo-EM model validation recommendations based on outcomes of the 2019 EMDataResource challenge
Source: Nat Methods. 2021 Feb 4;18(2):156–64. doi: 10.1038/s41592-020-01051-w (PMC7864804; doi:10.1038/s41592-020-01051-w)

nature portfolio

https://doi.org/10.1038/s41592-020-01051-w

Supplementary information

Cryo-EM model validation recommendations based on outcomes of the 2019 EMDataResource challenge

In the format provided by the authors and unedited

Supplementary Figure 1 for:

Cryo-EM model validation recommendations based on outcomes of the 2019 EMDataResource challenge

Catherine L. Lawson*, Andriy Kryshtafovych, Paul D. Adams, Pavel V. Afonine, Matthew L. Baker, Benjamin A Barad, Paul Bond, Tom Burnley, Renzhi Cao, Jianlin Cheng, Grzegorz Chojnowski, Kevin Cowtan, Ken A. Dill, Frank DiMaio, Daniel P. Farrell, James S. Fraser, Mark A. Herzik Jr., Soon Wen Hoh, Jie Hou, Li-Wei Hung, Maxim Igaev, Agnel P. Joseph, Daisuke Kihara, Dilip Kumar, Sumit Mittal, Bohdan Monastyrskyy, Mateusz Olek, Colin M. Palmer, Ardan Patwardhan, Alberto Perez, Jonas Pfab, Grigore D. Pintilie, Jane S. Richardson, Peter B. Rosenthal, Daipayan Sarkar, Luisa U. Schäfer, Michael F. Schmid, Gunnar F. Schröder, Mrinal Shekhar, Dong Si, Abishek Singharoy, Genki Terashi, Thomas C. Terwilliger, Andrea Vaiana, Liguo Wang, Zhe Wang, Stephanie A. Wankowicz, Christopher J. Williams, Martyn Winn, Tianqi Wu, Xiaodi Yu, Kaiming Zhang, Helen M. Berman, Wah Chiu*

Supplementary Figure 1. Model Score Distributions

Score distributions for all 63 models submitted to the challenge are shown on the following three pages (A: Fit-to-Map; B: Coordinates-only; C: Comparison-to-Reference). Parallel lanes display score distributions for each target and each evaluated metric. Scores are plotted horizontally as semi-transparent diamonds against a color-coded background to indicate worse (left, orange) and better (right, green) values. Where multiple scores overlap, the semi-transparent diamonds appear as a single darker, more opaque diamond. Reference model scores are indicated as red triangles.

A. Fit-to-Map

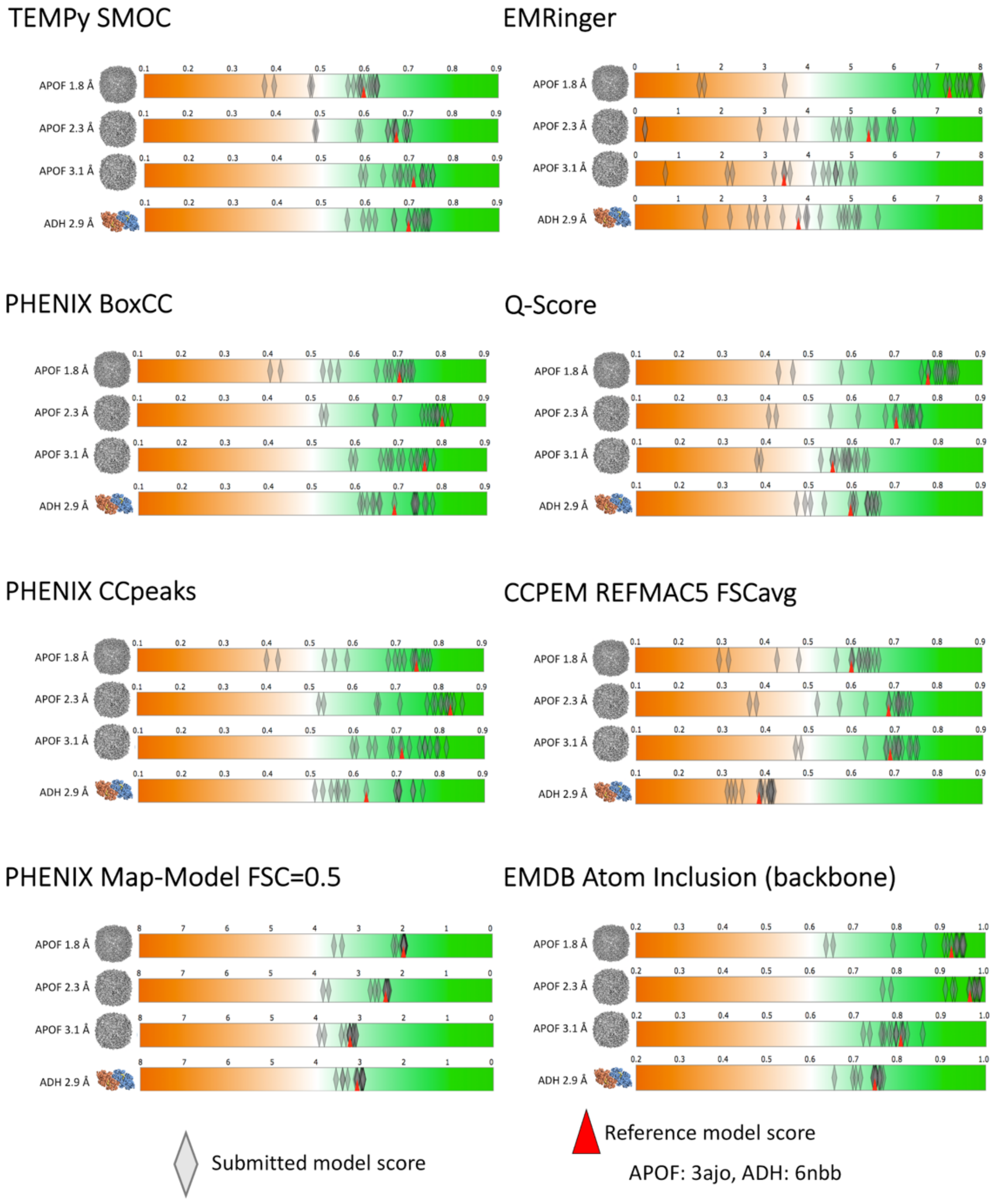
TEMPy SMOC
EMRinger
PHENIX BoxCC
Q-Score
PHENIX CCpeaks
CCPEM REFMAC5 FSCavg
PHENIX Map-Model FSC=0.5
EMDB Atom Inclusion (backbone)
APOF 1.8 Å
APOF 2.3 Å
APOF 3.1 Å
ADH 2.9 Å
Submitted model score
Reference model score
APOF: 3ajo, ADH: 6nbb

B. Coordinates-only

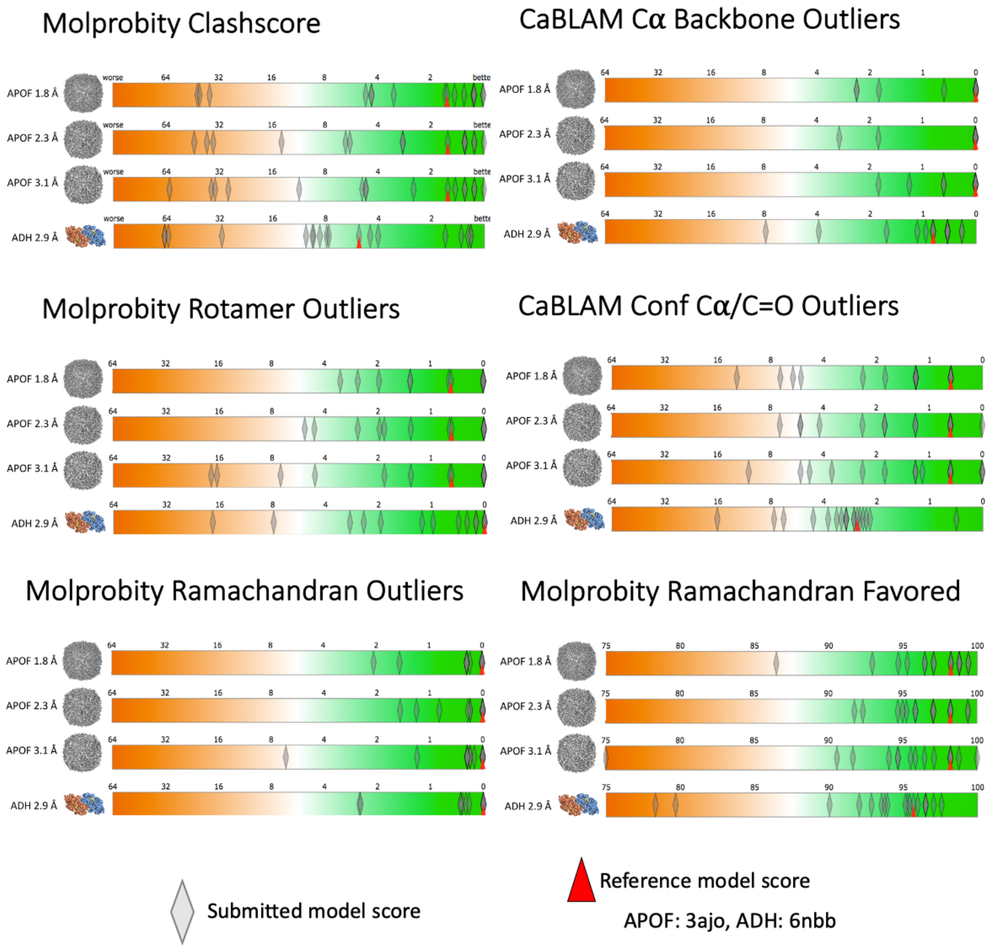

C. Comparison with Reference (monomeric)

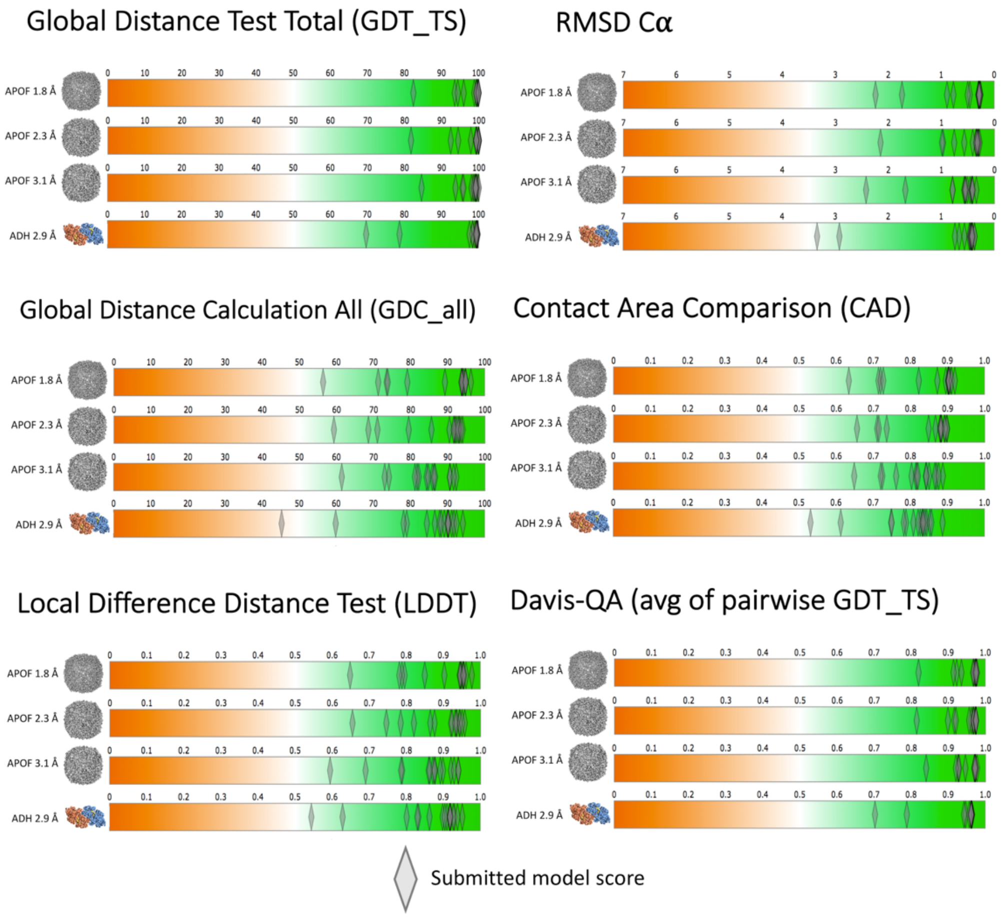

Supplement: Supplementary file 1 — Supplementary Fig. 1. [file 41592_2020_1051_MOESM1_ESM.pdf]
