# Supplementary material for: Cryo-EM model validation recommendations based on outcomes of the 2019 EMDataResource challenge
Source: Nat Methods. 2021 Feb 4;18(2):156–64. doi: 10.1038/s41592-020-01051-w (PMC7864804; doi:10.1038/s41592-020-01051-w)
Supplement: Supplementary file 2 — Reporting Summary [file 41592_2020_1051_MOESM2_ESM.pdf]

nature research

Corresponding author(s): Catherine L. Lawson, Wah Chiu

Last updated by author(s): Nov 12, 2020

Reporting Summary

Nature Research wishes to improve the reproducibility of the work that we publish. This form provides structure for consistency and transparency in reporting. For further information on Nature Research policies, see Authors & Referees and the Editorial Policy Checklist.

Statistics

For all statistical analyses, confirm that the following items are present in the figure legend, table legend, main text, or Methods section.

n/a	Confirmed	
☐	☒	The exact sample size (*n*) for each experimental group/condition, given as a discrete number and unit of measurement
☒	☐	A statement on whether measurements were taken from distinct samples or whether the same sample was measured repeatedly
☒	☐	The statistical test(s) used AND whether they are one- or two-sided<br>*Only common tests should be described solely by name; describe more complex techniques in the Methods section.*
☒	☐	A description of all covariates tested
☐	☒	A description of any assumptions or corrections, such as tests of normality and adjustment for multiple comparisons
☒	☐	A full description of the statistical parameters including central tendency (e.g. means) or other basic estimates (e.g. regression coefficient) AND variation (e.g. standard deviation) or associated estimates of uncertainty (e.g. confidence intervals)
☒	☐	For null hypothesis testing, the test statistic (e.g. *F*, *t*, *r*) with confidence intervals, effect sizes, degrees of freedom and *P* value noted<br>*Give P values as exact values whenever suitable.*
☒	☐	For Bayesian analysis, information on the choice of priors and Markov chain Monte Carlo settings
☒	☐	For hierarchical and complex designs, identification of the appropriate level for tests and full reporting of outcomes
☒	☐	Estimates of effect sizes (e.g. Cohen's *d*, Pearson's *r*), indicating how they were calculated

Our web collection on statistics for biologists contains articles on many of the points above.

Software and code

Policy information about availability of computer code

Data collection

Software for Models prepared by participating groups:

Ab initio
ARP/wARP v.8.0 arpwarp.embl-hamburg.de (groups 27, 41)
CCPEM v.1.2.0/Buccaneer-v.1.16.8* www.ccpem.ac.uk www.ccp4.ac.uk (group 27)
CCPEM v.1.3.0/Buccaneer-v.1.16.8* www.ccpem.ac.uk www.ccp4.ac.uk (groups 10, 28)
Cascaded-CNN v.1.0 github.com/DrDongSi/Ca-Backbone-Prediction (group 60)
Mainmast v.1.0 kiharalab.org/mainmast (groups 54, 73)
Pathwalker v.2.0 blake.bcm.edu/emanwiki/EMAN2/Programs/e2pathwalker (group 90)
Rosetta 3.9 rosettacommons.org (groups 27, 54, 82**)

optimization
CDMD v.gromacs-5.0.7-densfit www.mpibpc.mpg.de/grubmueller/densityfitting (group 25)
CNS v.1.3 cns-online.org/v1.3 (group 38)
DireX v.0.7.1 simtk.org/home/direx (group 38)
Gromacs v.2018.6 gromacs.org (group 38)
Phenix/real_space_refine v.1.15 phenix-online.org (groups 10, 27, 35, 38, 91)
CCPEM v.1.3.0/Refmac v.5.7* www.ccpem.ac.uk www.ccp4.ac.uk (groups 28, 41)
MELD 0.2.3 github.com/maccallumlab/meld (group 73)
MDFF v.0.4 www.ks.uiuc.edu/Research/vmd/plugins/mdff (groups 38, 54, 73
reMDFF v.0.4 github.com/jvant/ReMDFF_Singharoy_Group (group 73)

Visual evaluation/manual model improvement:
VMD v.1.9.3 www.ks.uiuc.edu/Research/vmd (groups 54, 73, 82)
UCSF Chimera v.1.11-v.1.14 www.cgl.ucsf.edu/chimera (groups 10, 38, 60, 73, 90)
PyMol v.2.2.0-v.2.3.0 github.com/schrodinger/pymol-open-source (groups 10, 27)

CCPEM/COOT v.1.3.0 www.ccpem.ac.uk (group 28)
COOT v.0.9-pre www2.mrc-lmb.cam.ac.uk/Personal/pemsley/coot (groups 10, 27, 28, 41, 90, 91)

Model coordinate submission metadata were collected using a Drupal webform.
Model coordinates were collected using pdb-extract.wwpdb.org and processed using MAXIT swtools.rcsb.org/apps/MAXIT

*The CCPEM package requires installation of the CCP4 package (www.ccp4.ac.uk) in order to run Buccaneer and Refmac.
**Full modeling scripts (group 82): https://faculty.washington.edu/dimaio/files/rosetta_em_challenge_2019.tgz

See also Table I

Data analysis

Fit-to-Map
TEMPy v.1.1 tempy.ismb.lon.ac.uk (CCC, CCC_OV, SMOC, LAP, MI, MI_OV, ENV)
Phenix/map_model_cc v.1.15 phenix-online.org (CCbox, CCpeaks, CCmask, FSC05)
Phenix/em_ringer v.1.15 phenix-online.org (EMRinger)
CCPEM v.1.4.1/ Refmac v.5.7* www.ccpem.ac.uk www.ccp4.ac.uk (FSCavg)
EMDB CryoEM Validation Analysis (va) v.0.0.dev8 pypi.org/project/va/0.0.0.dev8 (AI_all)

Coordinates-only
Phenix/molprobity v.1.15 phenix-online.org (CaBLAM, Clashscore, Rotamer, Rama, Calpha)
Phenix/model_statistics v. 1.15 phenix-online.org (Bond, Angle, Chiral, Planar, Dihedral)
MAPQ v.1.2 github.com/gregdp/mapq (Qscore)
KiNG 2.23 kinemage.biochem.duke.edu/software (issue visualization)

Comparison-to-Reference
LGA v.04.2019 proteinmodel.org/AS2TS/LGA/lga.html (GDT-TS, GDC, GDC-SC, DAVIS-QA)
OpenStructure/LDDT v.2.1 www.openstructure.org/download (LDDT)
CAD v.1646 bitbucket.org/kliment/voronota/src/master (CAD)
HBPLUS v.3.06 www.ebi.ac.uk/thornton-srv/software/HBPLUS (HBPR>6)

*The CCPEM package requires installation of the CCP4 package (www.ccp4.ac.uk) in order to run Refmac.

See also Online Methods and Table II.

For manuscripts utilizing custom algorithms or software that are central to the research but not yet described in published literature, software must be made available to editors/reviewers. We strongly encourage code deposition in a community repository (e.g. GitHub). See the Nature Research guidelines for submitting code & software for further information.

Data

Policy information about availability of data

All manuscripts must include a data availability statement. This statement should provide the following information, where applicable:

- Accession codes, unique identifiers, or web links for publicly available datasets
- A list of figures that have associated raw data
- A description of any restrictions on data availability

The map targets used in the Challenge were downloaded from EM Data Bank, entries:
EMD-20026 (file: emd_20026_additional_1.map.gz),
EMD-20027 (file: emd_20027_additional_2.map.gz),
EMD-20028, (file: emd_20028_additional_2.map.gz), and
EMD-0406. (file: emd_0406.map.gz)
Reference models were downloaded from Protein Data Bank, entries 3ajo and 6nbb.

Submitted models, model metadata, result logs, and compiled data are archived at Zenodo: https://doi.org/10.5281/zenodo.4148789, and at https://model-compare.emdataresource.org/data/2019/. Interactive summary tables, graphical views, and csv downloads of compiled results are available at https://model-compare.emdataresource.org/2019/cgi-bin/index.cgi.

Field-specific reporting

Please select the one below that is the best fit for your research. If you are not sure, read the appropriate sections before making your selection.

☒ Life sciences ☐ Behavioural & social sciences ☐ Ecological, evolutionary & environmental sciences

For a reference copy of the document with all sections, see nature.com/documents/nr-reporting-summary-flat.pdf

Life sciences study design

All studies must disclose on these points even when the disclosure is negative.

Sample size

Sample size was determined by the number of model coordinate submissions. Sample size was sufficient to meet the goal of qualitatively comparing model quality across current methods in use, and assessing usefulness of different model metrics. The Challenge was not designed to quantitatively and exhaustively explore all variables.

Data exclusions	All 63 submitted models were evaluated, with the exception that model hydrogen atom positions and refined B-factors were excluded from the reported Fit-to-Map analyses.
Replication	Participating groups were asked to complete the same four modeling tasks, yielding 15-17 models per task. Each model was created independently, so there are no exact replicates.
Randomization	Not applicable--No attempt was made to randomize the data.
Blinding	Initial evaluations of the submitted coordinates were blinded to the identity of the participating groups and software used.

Reporting for specific materials, systems and methods

We require information from authors about some types of materials, experimental systems and methods used in many studies. Here, indicate whether each material, system or method listed is relevant to your study. If you are not sure if a list item applies to your research, read the appropriate section before selecting a response.

Materials & experimental systems

n/a	Involved in the study
☒	☐ Antibodies
☒	☐ Eukaryotic cell lines
☒	☐ Palaeontology
☒	☐ Animals and other organisms
☒	☐ Human research participants
☒	☐ Clinical data

Methods

n/a	Involved in the study
☒	☐ ChIP-seq
☒	☐ Flow cytometry
☒	☐ MRI-based neuroimaging
